# Synthetic Sequencing Standards: A Guide to Database Choice for Rumen Microbiota Amplicon Sequencing Analysis

**DOI:** 10.3389/fmicb.2020.606825

**Published:** 2020-12-08

**Authors:** Paul E. Smith, Sinead M. Waters, Ruth Gómez Expósito, Hauke Smidt, Ciara A. Carberry, Matthew S. McCabe

**Affiliations:** ^1^Teagasc Animal and Bioscience Research Department, Teagasc Grange, Meath, Ireland; ^2^UCD School of Agricultural and Food Science, University College Dublin, Dublin, Ireland; ^3^Laboratory of Microbiology, Wageningen University & Research, Wageningen, Netherlands

**Keywords:** rRNA, amplicon sequencing, rumen microbiota, sequencing standard, reference database

## Abstract

Our understanding of complex microbial communities, such as those residing in the rumen, has drastically advanced through the use of high throughput sequencing (HTS) technologies. Indeed, with the use of barcoded amplicon sequencing, it is now cost effective and computationally feasible to identify individual rumen microbial genera associated with ruminant livestock nutrition, genetics, performance and greenhouse gas production. However, across all disciplines of microbial ecology, there is currently little reporting of the use of internal controls for validating HTS results. Furthermore, there is little consensus of the most appropriate reference database for analyzing rumen microbiota amplicon sequencing data. Therefore, in this study, a synthetic rumen-specific sequencing standard was used to assess the effects of database choice on results obtained from rumen microbial amplicon sequencing. Four DADA2 reference training sets (RDP, SILVA, GTDB, and RefSeq + RDP) were compared to assess their ability to correctly classify sequences included in the rumen-specific sequencing standard. In addition, two thresholds of phylogenetic bootstrapping, 50 and 80, were applied to investigate the effect of increasing stringency. Sequence classification differences were apparent amongst the databases. For example the classification of *Clostridium* differed between all databases, thus highlighting the need for a consistent approach to nomenclature amongst different reference databases. It is hoped the effect of database on taxonomic classification observed in this study, will encourage research groups across various microbial disciplines to develop and routinely use their own microbiome-specific reference standard to validate analysis pipelines and database choice.

## Introduction

The ability of ruminant animals to obtain nutrition from complex plant carbohydrates stems from their co-evolution with the microbial community residing in their rumen (Sasson et al., 2017). Traditionally, the rumen microbiome was investigated using culture based methods such as those pioneered by Hungate (1966). Unfortunately, such methods were often biased toward the study of culturable microbes (Huws et al., 2018) and left an estimated 80–90% of the rumen microbiome undiscovered (McSweeney et al., 2006). However, the widespread availability of high throughput sequencing (HTS) technology after the turn of the 21st century has advanced our understanding of numerous complex microbial communities, including that which inhabits the rumen (Knight et al., 2018).

Metabarcoded amplicon sequencing has become the most popular method for studying the microbial composition of a variety of microbiomes (Pollock et al., 2018) with numerous authors using it to study the relationship of the rumen microbial community with dietary management (Petri et al., 2013; McCabe et al., 2015; Lyons et al., 2017; Smith et al., 2020) host genetics (Henderson et al., 2015) enteric methane output (Kittelmann et al., 2014; Danielson et al., 2017) and production traits such as feed efficiency (Myer et al., 2015; McGovern et al., 2018a) or milk production (Jami et al., 2014). The method can be described as the targeted PCR amplification of a specific gene or genomic segment of interest (amplicon), with primers targeting conserved regions which flank regions of variability (Allaband et al., 2019). Popular phylogenetic marker genes used to investigate microbial communities include; 16S ribosomal RNA (rRNA) (bacteria and archaea), 18S rRNA (protozoa) and the internal transcribed spacer region (fungi).

The method is championed for its ability to produce large amounts of relatively inexpensive microbial composition data, be predominantly free from eukaryotic host contamination and with downstream data processing and analysis that is less computationally demanding in comparison to other methods, such as shotgun metagenomics (Knight et al., 2018; Fricker et al., 2019). In addition, the technique has previously been utilized in large scale global studies such as the Earth Microbiome Project (Thompson et al., 2017) and the Global Rumen Census (GRC) project (Henderson et al., 2015). Unlike shotgun metagenomics, 16S rRNA gene amplicon sequencing benefits from the availability of extensive and comprehensive curated reference databases for the alignment of sequences or training of taxonomic classifiers, and ultimately taxonomic classification of sequences. The most widely available reference databases include the Ribosomal Database Project (RDP) (Cole et al., 2014), SILVA (Quast et al., 2012), GreenGenes (DeSantis et al., 2006), RefSeq (O’Leary et al., 2016) and, more recently, the Genome Taxonomy Database (GTDB) (Parks et al., 2018). In addition, the Rumen and Intestinal Methanogen Database (RIM-DB) is a methanogen specific reference database (Seedorf et al., 2014).

The calibration of high throughput analysis with an internal control or reference standards is common practice in many scientific disciplines (Hardwick et al., 2017; Yeh et al., 2018). However, in the area of molecular microbiology, the use of internal reference standards to “benchmark” results and analysis remains low. For example, of the 265 high throughput microbial experiments published in the journals Microbiome and ISME in 2018, only 10% of studies reported the use of internal positive controls (Hornung et al., 2019).

The effect of reference database choice on the classification of sequences and the implications this has on the interpretation of microbiota analysis is well established (Park and Won, 2018; Pollock et al., 2018; Henderson et al., 2019). However, few detailed comparisons between the classification accuracy of the main reference databases, with a rumen microbiota focus, have been conducted with the use of a reference standard. The dominance of microbial data originating from human microbiomes in many databases (Pollock et al., 2018) has resulted in little consensus surrounding the choice of the optimal 16S rRNA database for rumen microbiome analysis. In light of this, the incorrect classification of rumen microbial generated sequences, when aligned to databases populated with predominantly human microbiome data, is a real concern. For example, sequences originating from the GRC have previously been shown to be both under estimated in, and impacted by, reference database choice (Henderson et al., 2019). In addition, the degree of bootstrapping applied during the bioinformatics analysis of amplicon data, has been shown to affect the accuracy of taxonomic classification. Bootstrapping is a technique which is applied to determine a threshold for the accuracy with which a sequence is classified (Lan et al., 2012) with an increase in the bootstrapping value from 50 to 80, previously shown to increase the accuracy of genus level classification (Claesson et al., 2009).

In accordance with the recommendations of the Microbiome Quality Control (MBQC) project and other research consortia, on the use of internal reference standards that contain taxa relevant to the microbial community under investigation (Sinha et al., 2015; Sinha et al., 2017; Pollock et al., 2018), the present study aimed to assess the accuracy of four reference databases (RDP, SILVA, GTDB and RefSeq + RDP) for rumen microbiota analysis. A synthetic amplicon reference standard containing a mixture of full length synthetic 16S and 18S rRNA gene sequences of key rumen microbes (archaea, bacteria and protozoa), was generated as part of the RumenPredict consortium and used in this study. In addition, this study aimed to determine the specificity of the Caporaso et al. (2011) 16S rRNA gene targeted primers to prokaryotic sequences and the effects of applying an enhanced degree of bootstrapping in the DADA2 pipeline on taxonomic classification.

## Materials and Methods

The full length 16S rRNA gene sequences of 13 different species of bacteria and 3 different species of archaea, identified as representative of the rumen microbial composition from the GRC project or prior studies and isolated from the rumen with their genome sequenced where possible, were synthesized based on sequence information obtained from GenBank by Integrated DNA Technologies (Leuven, Belgium). Species were deemed representative of the rumen microbial community by being several of the most abundant classified genera in the GRC project, as well as species known to be commonly associated with different diets fed to domesticated ruminants. In addition, nine rumen protozoal 18S rRNA gene sequences (synthesized in the same manner as the 16S rRNA gene sequences) were present in the reference standard to evaluate the specificity of the 16S rRNA gene primers utilized. Details of the full length 16S (*n* = 16) and 18S (*n* = 9) rRNA gene sequences included in the reference standard are highlighted in Table 1. Synthesized sequences were pooled at varied concentrations to create the standard. The standard was treated as an internal control and included in three separate rumen microbial amplicon sequencing runs, as part of additional studies, with library preparation identical for all three runs. The standard was subsequently sequenced once per run, resulting in three sequenced replicates of the standard for comparison.

**TABLE 1:** Classification of full length 16S (bacteria and archaea; *n* = 16) and 18S (protozoa; *n* = 9) rRNA sequences included in the rumen specific reference standard.

**Sequence**	**Classification**
1	Eubacterium_ruminantium_strain_GA195_NR_024661.1_Isolated_from_bovine_rumen_1508bp
2	Methanobrevibacter_millerae_strain_SM9_CP011266.1:785177-786613_Isolated_from_rumen_fluid_of_Ovis aries_1437bp
3	Methanobrevibacter_olleyae_strain_YLM1_CP014265.1:274144-275584 _Isolated from_rumen_content_of_Ovis_aries_1441bp
4	Peptostreptococcus_anaerobius_ATCC_27337_L04168.1_Isolated_from_bovine_rumen_1462bp
5	Pseudobutyrivibrio_ruminis_HUN009_NZ_JNLH01000001.1:24802-26343_Isolated_from_rumen_1542bp*
6	Propionibacterium_ruminibrarum_DSM106771T_Isolated_from_cow_rumen_fibrous_content_1430bp
7	Ruminococcus_albus_strain_7_CP002403.1:417348-418847_Isolated_from_cow rumen_1500bp
8	Butyrivibrio_fibrisolvens_ATCC_19171_U41172.1_Isolated_from_bovine_rumen_1477bp
9	Megasphaera_elsdenii_strain_DSM_20460_(= ATCC_25940)_NR_102980.1_Isolated_from_sheep_rumen_1552bp
10	Methanogenic_archaeon_strain_ISO4-H5 Methanomassiliicoccales_NZ_ CP014214.1:1550802-1552276_ Isolated from_rumen_content_of_Ovis_aries 1475bp
11	Streptococcus_bovis_strain_B315_AF396920.1_Isolated_from_cattle_rumen_1540bp
12	Lachnospira_multipara_DSM3073T_Isolated_from_bovine_rumen_1453bp
13	Fibrobacter_succinogenes_subsp._succinogenes_S85_NC_017448.1:1232471-1233969_Isolated_from_bovine_rumen_1499bp
14	Clostridium_aminophilum_strain_F_NR_118651.1_Isolated_from_bovine_rumen_1477bp
15	Selenomonas_ruminantium_strain_GA192_M62702.1_Isolated_from_bovine_rumen_1367bp
16	Prevotella_ruminicola_strain_23_NC_014033.1:223907-225439_Isolated_from_ rumen_1533bp
17	Entodinium_caudatum_U57765.1_18S_Isolated_from_sheep_rumen_18S_1639bp
18	Epidinium_ecaudatum_caudatum_AM158474.1_Isolated_from_sheep_rumen_18S_1637bp
19	Ostracodinium_gracile_AM158468.1_Isolated_from_cattle_rumen_18S_1639bp
20	Isotricha_prostoma_AF029762.1_Isolated_from_cow_rumen_18S_1641bp
21	Dasytricha_ruminantium_U57769.1_Isolated_from_sheep_rumen_18S_1638bp
22	Eudiplodinium_maggii_U57766.1_Isolation_from_rumen_18S_1637bp
23	Diploplastron_affine_AM158457.1_Isolated_from_cattle_rumen_18S_1627bp
24	Metadinium_medium_AM158464.1_Isolated_from_cattle_18S_1627bp
25	Polyplastron_multivesiculatum_AM158458.1_Isolated_from_sheep_rumen_18S_1629bp

Sequence data and classifications were sourced from GenBank. *16S sequence of *Pseudobutyrivibrio ruminis* obtained from a draft genome.

### Library Preparation and Sequencing

Illumina amplicon libraries were generated using 12.5 ng of synthetic DNA from the pooled rumen sequencing standard as a template. Two rounds of PCR amplification were performed according to instructions in the Illumina MiSeq 16S Sample Preparation Guide with minor modifications to cycle length, as previously described by McGovern et al. (2018b). The first round of PCR amplification was performed using the 515F/806R primers (Caporaso et al., 2011), targeting the V4 hypervariable region of the 16S rRNA gene. These primers have previously been utilized for the amplification of bacterial and archaeal microbial communities in large scale studies, such as the Earth Microbiome Project (Thompson et al., 2017) and investigations of the prokaryotic microbial community residing in the rumen (McCabe et al., 2015; Bowen et al., 2018; Petri et al., 2018; McGovern et al., 2018a; McLoughlin et al., 2020; Smith et al., 2020). In addition, primers were designed with Illumina Nextera overhang adapters, with the 2× KAPA Hifi HotStart ReadyMix DNA polymerase (Roche Diagnostics, West Sussex, United Kingdom) utilized during amplification. Cycle conditions were as follows: 1 cycle of 95°C for 3 min, then 20 cycles of 95°C for 30 s, 55°C for 30 s, 72°C for 30 s and then 1 cycle of 72°C for 5 min.

Amplicons were purified using the MinElute PCR Purification Kit (Qiagen, Manchester, United Kingdom). Following purification, amplicons were subjected to a second round of PCR to attach dual indices and Illumina sequencing adapters using the Nextera XT indexing kit (Illumina, San Diego, CA, United States). Cycle conditions for the second round of PCR were 1 cycle of 95°C for 3 min, then 8 cycles of 95°C for 30 s, 55°C for 30 s, 72°C for 30 s and then 1 cycle of 72°C for 5 min. The resulting amplicons were then purified using the MinElute PCR Purification Kit (Qiagen, Manchester, United Kingdom). Amplicon size was assessed by electrophoresis on a 2% agarose gel. Amplicons generated from the synthetic standard were pooled in equal concentration to the rumen microbial amplicons specific to each run and subjected to gel purification using the QIAquick Gel Extraction Kit (Qiagen, Manchester, United Kingdom) to remove adapter dimers. An additional round of purification with a MinElute PCR purification kit (Qiagen, Manchester, United Kingdom) was then conducted to remove any residues of agarose.

Pooled sample purity and quantity was analyzed on a Nanodrop 1000. Quantification was also performed on a Qubit fluorometer and using the KAPA SYBR FAST universal kit with Illumina Primer Premix (Roche Diagnostics, West Sussex, United Kingdom). Following this, the library pool was diluted and denatured according to the Illumina MiSeq 16S Sample Preparation Guide. Sequencing was conducted on three separate occasions on the Illumina MiSeq using 500 cycle (version 2) reagent kits (Illumina, San Diego, CA, United States).

### Sequencing Analysis

All sequences were processed in R (version 3.5.2) using DADA2 (version 1.11.3)^1^ with each of the three MiSeq runs separately submitted to the DADA2 pipeline as described by Callahan et al. (2016) with minor modifications. Quality checks were conducted to ensure that forward and reverse reads had mean Q scores of >30. Guided by the mean Q scores, forward reads were trimmed to a length of 240 bp and reverse reads trimmed to 200 bp. Primer sequences were removed using the trimLeft function. Identical sequences were combined using the dereplication function followed by the merging of forward and reverse reads. Following this, an amplicon sequence variant (ASV) table was generated and chimeric sequences were removed.

The performance of four databases, generated for use in the DADA2 pipeline, was assessed. Taxonomic assignment of sequence variants was compared using SILVA (version 132), Ribosomal Database Project (RDP; version 11.5), Genome Taxonomy Database (GTDB; release date 20/11/2018) and the RefSeq + RDP (release date 14/05/2018) available for download from the DADA2 website^2^. Both GreenGenes and the RIM-DB were omitted from our analysis due to the classification of sequences based on predefined levels of similarity or grouping of species into phylogenetic clades (DeSantis et al., 2006; Seedorf et al., 2014). All four databases represent training data sets formatted for use with the RDP Naïve Bayesian classifier within DADA2. Taxonomy was assigned to the RDP and SILVA database using a combination of the functions assignTaxonomy and assignSpecies with the appropriate taxonomy and species formatted fasta files. Only the assignTaxonomy function was used for both the GTDB and RefSeq + RDP databases as they contain species level classifications. Bootstrapping was applied at the default threshold implemented in DADA2 of 50 and 80. The increased level of bootstrapping was applied with the addition of the minBoot = 80 function to all assignTaxonomy and assignSpecies functions (see Supplementary Data 1).

Sample metadata, sequence taxonomy generated for each database and ASVs were combined into a phyloseq object for each run using phyloseq (version 1.24.2) (McMurdie and Holmes, 2013). Individual phyloseq objects for each database, across the three runs, were combined using the merge_phyloseq function to facilitate database comparisons. All ASVs which did not align to bacterial and archaeal sequences were depleted from all databases. However, to assess primer specification, all sequences were retained in an additional set of SILVA ASV tables. For database comparisons, two ASV tables, to account for the different bootstrapping thresholds, were generated per database (i.e., a total of *n* = 8 tables), with each table containing data from the all sequencing runs (*n* = 3). An additional two ASV tables, accounting for both bootstrapping values, were compiled for SILVA for the aforementioned primer specification analysis. In total, ten ASV tables were generated for analysis.

The generated ASV table and sequence taxonomies for each database were analyzed in R (version 3.5.2). Spearman’s rank correlation coefficient, generated with the hmisc package (version 4.2.0), was used to evaluate the compositional consistency of the standard across the three sequencing runs. The relative abundance of taxa was calculated for each sample at the species level in phyloseq.

### Analysis of Database Classification and Primer Specification

The performance of each database was primarily assessed on their ability to correctly assign each of the 16S rRNA sequences, from kingdom to the correct genus and species level, with the exception of Methanomassiliicoccales which is yet to be classified beyond the taxonomic level of order (Li et al., 2016). The classification of the 16 most abundant sequences in each ASV table was utilized in the database comparison, with additionally populated sequences considered as background. The 16 most abundant ASVs were selected for analysis, based on the number of bacterial (*n* = 13) and archaeal (*n* = 3) sequences included in the standard. The mean relative abundance of each classified microbial sequence across the three runs was used for comparisons. Classification of each of the 16 most abundant ASV sequences was confirmed by conducting an online nr/nt NCBI BLAST search^3^.

Primer specificity was investigated, using only the SILVA (version 132) database, by determining the proportion of sequences assigned to prokaryotic and eukaryotic kingdoms. All non-classified and eukaryotic sequences were assessed as chimeric using the online version of DECIPHER (version 2.17.1^4^) (Wright et al., 2012). The classification of non-chimeric sequences was confirmed using the online nr/nt NCBI BLAST as previously mentioned.

## Results

The reference standard was included once across three separate sequencing runs, resulting in three sequenced replicates of the standard for comparison. The expected and actual genus and species level classification of bacterial (*n* = 13) and archaeal (*n* = 3) sequences included in the reference standard, by the four databases, are displayed in Table 2 (50 bootstraps) and Table 3 (80 bootstraps). A more detailed description of all levels of taxonomic classification for each database can be found in the Supplementary Materials (see Supplementary Tables 1, 2).

**TABLE 2:** Expected vs. actual classification of bacterial (*n* = 13) and archaeal (*n* = 3) sequences included in reference standard by four databases at a bootstrapping threshold of 50.

**Sequence**	**Expected Classification**	**GTDB**	**RDP**	**SILVA**	**RefSeq + RDP**
1	*Eubacterium ruminantium*	FF	FF	FF	UE
2	*Methanobrevibacter millerae*	EE	EF	EF	EE
3	*Methanobrevibacter olleyae*	EE	EF	EF	EE
4	*Peptostreptococcus anaerobius*	FF	EE	EE	EE
5	*Pseudobutyrivibrio ruminis*	EE	EF	EF	EE
6	*Propionibacterium sp.*	FF	EF	EU	EU
7	*Ruminococcus albus*	EE	EE	EE	EE
8	*Butyrivibrio fibrisolvens*	FF	EE	EE	EE
9	*Megasphaera elsdenii*	EE	EF	EF	EF
10	*Methanogenic archaeon strain ISO4-H5 Methanomassiliicoccales*	UU	UF*	UF*	UU
11	*Streptococcus bovis*	EU	EF	EF	EU
12	*Lachnospira multipara*	EU	EF	EF	EE
13	*Fibrobacter succinogenes*	EE	EE	EE	EE
14	*Clostridium aminophilum*	FF	UF	UF	EE
15	*Selenomonas ruminantium*	EE	EE	EE	EE
16	*Prevotella ruminicola*	EF	EE	EE	EE

F, database failed to assign ASV to taxonomic level. E, database assigned ASV to the expected taxonomic level. U, database assigned ASV to the unexpected taxonomic level.*Both the RDP and SILVA database gave a genus, but not a species, level classification.

**TABLE 3:** Expected vs. actual classification of bacterial (*n* = 13) and archaeal (*n* = 3) sequences included in reference standard by four databases at a bootstrapping threshold of 80.

**Sequence**	**Expected Classification**	**GTDB**	**RDP**	**SILVA**	**RefSeq +RDP**
1	*Eubacterium ruminantium*	FF	FF	FF	UE
2	*Methanobrevibacter millerae*	EE	EF	EF	EE
3	*Methanobrevibacter olleyae*	EE	EF	EF	EE
4	*Peptostreptococcus anaerobius*	FF	EE	EE	EE
5	*Pseudobutyrivibrio ruminis*	EE	EF	EF	EE
6	*Propionibacterium sp.*	EF	EF	EU	EE
7	*Ruminococcus albus*	EE	EE	EE	EE
8	*Butyrivibrio fibrisolvens*	FF	EE	EE	EE
9	*Megasphaera elsdenii*	EE	EF	EF	EF
10	*Methanogenic archaeon strain ISO4-H5 Methanomassiliicoccales*	UU	UU*	UU*	EE**
11	*Streptococcus bovis*	EU	EF	EF	EU
12	*Lachnospira multipara*	EU	EF	EF	EE
13	*Fibrobacter succinogenes*	EE	EE	EE	EE
14	*Clostridium aminophilum*	FF	UF	UF	EE
15	*Selenomonas ruminantium*	EE	EE	EE	EE
16	*Prevotella ruminicola*	EF	EE	EE	EE

*F, database failed to assign ASV to taxonomic level. E, database assigned ASV to the expected taxonomic level. U, database assigned ASV to the unexpected taxonomic level. ^∗^Both the RDP and SILVA database gave a genus, but not a species, level classification. ^∗∗^RefSeq + RDP database was unable to classify ASV beyond phylum.*

### Sequencing Performance

Across the three sequencing runs, an average of 246,721 ± 69,789 reads was obtained for the rumen-specific sequencing standard. Following quality filtering, merging and removal of chimeric sequences, the average number of reads for the sequencing standard was 207,816 ± 46,679 reads per run. Four applications of the DADA2 pipeline, each time with either the SILVA, RDP, RefSeq + RDP or GTDB databases, resulted in the identification of the expected 16 ASVs (each with a relative abundance of >1.0%), which represented ∼99.5% of all the ASVs identified across the sequencing runs. The remaining ASVs (representing just 0.5% of all the sequences) had a relative abundance of <1.0%. An average correlation, over the course of the three runs, of r_*s*_ 0.90 was observed in the composition of the 16 most abundant microbes included in the sequencing standard, across all four databases.

### Domain Level Primer Specificity

Of the four databases analyzed, SILVA is the only database which contains 18S rRNA reference sequences and hence was used for the determination of the prokaryotic specificity of the 515F/806R primers (Caporaso et al., 2011). A total of 61 unique ASVs were identified using the SILVA database when sequences from all kingdoms in the SILVA database were included. The 16 most abundant phylotypes classified by the SILVA database, on average, had a combined relative abundance of 99.48 ± 0.003% and were identified as bacterial and archaeal ASVs only. A total of 12 ASVs were classified as eukaryotic, when a bootstrapping threshold of 50 was initiated, and had a mean relative abundance of <0.02%. The number of ASVs identified as eukaryotic decreased to nine when a bootstrapping cut off of 80 was applied, with the remaining three ASVs unclassified to any kingdom. BLAST search (nr/nt) confirmed only one of these 12 ASVs to be truly eukaryotic (mean relative abundance < 0.001%). Of the remaining 11 ASVs, one ASV was deemed chimeric (mean relative abundance 0.004%) with the remaining ten ASVs identified as bacteria or archaea from the top nr/nt NCBI BLAST hits.

### GTDB Database

**FIGURE 1:**
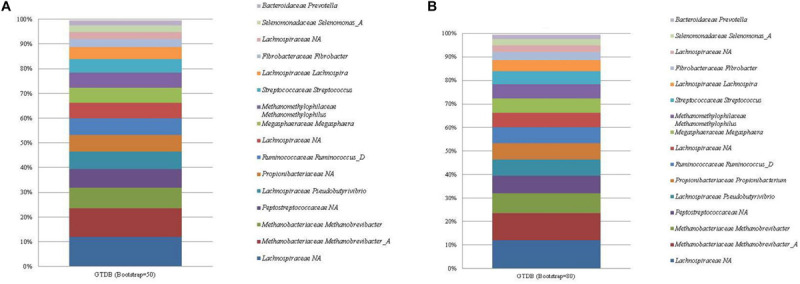
The mean relative abundance and GTDB classification (family and genus) of the 16 most abundant sequences in libraries generated from the rumen specific reference standard. Bootstrapping threshold: **(A)** 50 and **(B)** 80.

The GTDB database classified 61 unique taxa with the 16 most abundant ASVs having a total relative abundance of 99.48%. An unclassified *Lachnospiraceae* (mean relative abundance of 11.95%) made up the largest proportion of ASVs identified by the GTDB database, regardless of bootstrapping threshold (Figure 1; see Supplementary Table 3). The ASVs associated with the unclassified *Lachnospiraceae* taxonomic assignment were confirmed as *E. ruminantium* via a BLAST nr/nt search. *Prevotella*, *Selenomonas*, and *Megasphaera* were classified to the correct genera with corresponding family level classifications of *Bacteroidaceae*, *Selenomonadaceae*, and *Megasphaeraceae*, respectively. In addition, ASVs classified as *Prevotella*, *Fibrobacter*, and *Propionibacterium* had corresponding phylum level classifications identified as *Bacteroidota*, *Fibrobacterota*, and *Actinobacteriota. P. anaerobius*, *C. aminophilum*, and *B. fibrisolvens* could not be classified beyond the family level. Finally, bootstrapping threshold impacted the classification of *Propionibacterium*, which could not be classified beyond the family level at the default cut off of 50.

Two ASVs were correctly classified as the *Methanobrevibacter* genus and species *M. olleyae* and *M. millerae*. Both *Methanobrevibacter* ASVs were classified to the phylum *Euryarchaeota*. The ASV assigned to Methanomassiliicoccales was classified to the correct taxonomic order. However, further attempts were made, irrespective of bootstrapping threshold, to classify the ASV to a lower taxonomic rank beyond the level of order to *Methanomethylophilus alvus*.

### SILVA and RDP Database

**FIGURE 2:**
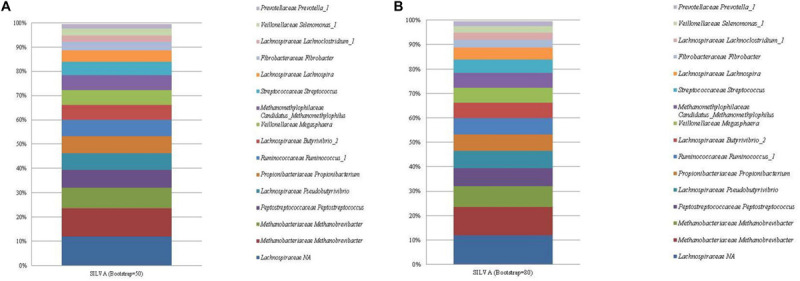
The mean relative abundance and SILVA classification (family and genus) of the 16 most abundant sequences in libraries generated from the rumen specific reference standard. Bootstrapping threshold: **(A)** 50 and **(B)** 80.

**FIGURE 3:**
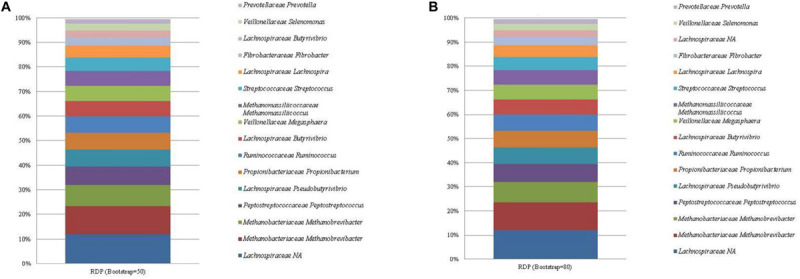
The mean relative abundance and RDP classification (family and genus) of the 16 most abundant sequences in libraries generated from the rumen specific reference standard. Bootstrapping threshold: **(A)** 50 and **(B)** 80.

Both SILVA and the RDP database performed similarly with respect to taxonomic identification, and resulted in the identification of a total of 49 and 59 unique ASVs, respectively. The most abundant ASV identified by both databases (Figures 2, 3; see Supplementary Tables 4, 5) was an unclassified sequence belonging to the family *Lachnospiraceae* (mean relative abundance of 11.96%). The genus level classifications of 11 of the 13 bacterial sequences included in the standard were similar across both databases. For the SILVA database, the remaining two bacterial sequences were assigned to the taxa *Lachnoclostridium* and the highly abundant *Lachnospiraceae*. NCBI nr/nt BLAST of these two ASVs generated *E. ruminantium* as the top hit for the highly abundant *Lachnospiraceae* and *C. aminophilum* as the top hit for *Lachnoclostridium*. No effect of bootstrapping was observed on ASVs classified with SILVA.

Similarly, the RDP database failed to classify the highly abundant ASVs assigned to *Lachnospiraceae* to a lower taxonomic rank. These ASVs generated *E. ruminantium* as the top hit when subjected to a BLAST nr/nt search. Bootstrapping threshold altered the classification of the *C. aminophilum* sequence. This ASV was classified as *Butyrivibrio* at the lower bootstrapping threshold but could not be classified beyond the level of family when bootstrapping was increased to 80.

Two ASVs were correctly classified as *Methanobrevibacter* within both databases. The methanogen Methanomassiliicoccales was classified to the correct taxonomic order, however, both RDP and SILVA further classified Methanomassiliicoccales as *Methanomassiliicoccus* and *Candidatus Methanomethylophilus*, respectively.

### RefSeq + RDP Database

**FIGURE 4:**
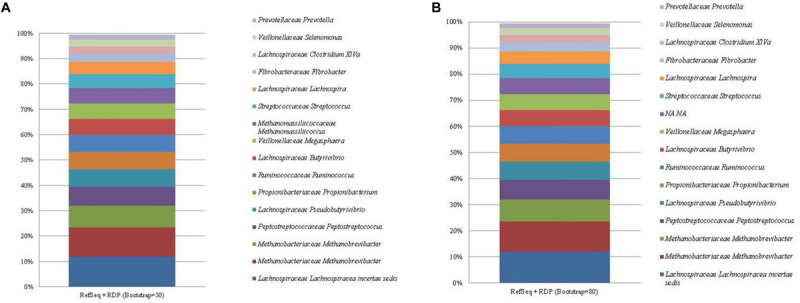
The mean relative abundance and RefSeq + RDP classification (family and genus) of the 16 most abundant sequences in libraries generated from the rumen specific reference standard. Bootstrapping threshold: **(A)** 50 and **(B)** 80.

The RefSeq + RDP database identified a total of 59 and 60 unique taxa at the high and low bootstrapping thresholds, respectively. The most abundant ASV (mean relative abundance of 11.96%) was identified as *Lachnospiraceae incertae sedis* at the genus level (Figure 4; see Supplementary Table 6). However, this sequence was classified as *E. ruminantium (AB008552)* at the species level (see Supplementary Tables 1, 2). The bacterial sequence *C. aminophilum* was given the genus level classification of *Clostridium XlVa*, however, was identified as *C. aminophilum (L04165)* at the species level (see Supplementary Tables 1, 2). All other remaining bacterial sequences were classified to the correct genus level. Bootstrapping threshold did not affect the taxonomic classification of any bacterial sequences to the genus level. However, at a threshold of 50, the *Propionibacterium* ASV was given a species level classification of *P. australiense* which was absent at the higher level of bootstrapping.

For the methanogen proportion of the standard, the RefSeq + RDP database correctly distinguished *Methanobrevibacter* as two separate ASVs. The degree of bootstrapping applied had an effect on the classification of the Methanomassiliicoccales sequence. When the default threshold of bootstrapping was applied, Methanomassiliicoccales was classified to the correct taxonomic order, however, similar to the other databases, this ASV was further classified beyond the taxonomic level of order as *Methanomassiliicoccus luminyensis (HQ896499)*. Methanomassiliicoccales was not classified beyond the taxonomic level of class when bootstrapping was increased to 80.

## Discussion

The importance of the routine inclusion of an internal DNA reference standard in sequencing runs should be apparent to all from the work of Yeh et al. (2018), who discovered the unexplainable disappearance of a key marine archaea from their internal control in one sequencing run. In spite of this, the routine inclusion and/or reporting of positive control use across microbial experiments remains low (Hornung et al., 2019).

In this study, using a rumen specific reference standard, we aimed to further validate the specificity of the 515F/806R primers (Caporaso et al., 2011) toward the prokaryotic domains, optimize our bioinformatics analysis and compare reference databases for rumen microbial 16S rRNA gene amplicon sequencing experiments. Based on the low (<0.001%) occurrence of truly non-prokaryotic taxa in the ASV table generated with the SILVA database, we can further confirm the suitability of the 515F/806R primers for investigations of the rumen bacterial and archaeal populations. Anaerobic fungi, while an important member of the rumen microbial community, were not included in this study as they are very much under-represented in the presently available microbial reference databases, due to the lack of fungal genome assemblies (Edwards et al., 2017; Huws et al., 2018). As a result, we did not seek to evaluate the precision with which each database would classify fungal sequences.

The DADA2 program implements the RDP Naïve Bayesian classifier, as described by Wang et al. (2007), for the taxonomic classification of sequences. A bootstrapping threshold of 50 is the default setting within DADA2 for taxonomic classification. A bootstrapping value of 80 has been shown to marginally increase the accuracy of genus level classification of sequences when targeting the V4 region of the 16S rRNA gene, but decrease the overall number of genus level classifications (Claesson et al., 2009). Increasing the bootstrapping threshold resulted in a more accurate classification of *Propionibacterium* in both the GTDB and RefSeq + RDP databases. At a bootstrapping level of 80, the incorrect species classification of *P. australiense* (Vaidya et al., 2019) was removed in the RefSeq + RDP along with the correct genus assignment of this ASV in the GTDB database. Furthermore, a more tentative classification of Methanomassiliicoccales was observed at the higher bootstrapping threshold in the RefSeq + RDP database. Therefore, to ensure the optimum level of accuracy from this point on, database comparison will only be discussed in terms of the datasets with which the higher level (80) of bootstrapping was applied.

Both the RDP and SILVA database have been routinely used in many microbiome experiments. The majority of 16S rRNA gene data for the RDP is generated from the International Nucleotide Sequence Database (INSC) (Cole et al., 2014). Sequence data within the SILVA database is obtained from EMBL-ENA (Glöckner et al., 2017). Both databases source taxonomy from versions of Bergey’s manual and are supplemented by the List of Prokaryotic Names with Standing in Nomenclature (LPSN) (Parte, 2018). In addition, the SILVA database supplements its taxonomic classification from the RDP (Glöckner et al., 2017) with rumen specific taxonomies added to recent versions of the SILVA database (Henderson et al., 2019).

The classification of a single ASV differed between both the RDP and SILVA databases. Previously, Henderson et al. (2019) observed a greater number of genera classification with SILVA, in comparison to RDP, with data generated as part of the GRC. Interestingly, SILVA identified the *C. aminophilum* sequence to the genus *Lachnoclostridium*. Previously it has been proposed to redefine all members of *Clostridium cluster XIVa* to the genus *Lachnoclostridium* (Yutin and Galperin, 2013). However, the nomenclatural *Lachnoclostridium* is described as a “preliminary entry that lacks crucial information” by the LPSN^5^. The RefSeq + RDP database classified this ASV as *Clostridium XIVa* and *C. aminophilum* at the genus and species level. Indeed, differences between the two databases showcase the need for inter database nomenclature consistency and alignment. In addition, this observation highlights further the effect database choice can have on sequence classification and importance of the routine reporting of database version to account for time specific changes to nomenclature.

The RefSeq database is curated from genomic data obtained and assessed by the National Centre for Biotechnology Information (NCBI) (O’Leary et al., 2016). In the analysis conducted by Balvoèiûtë and Huson (2017), the number of 16S rRNA gene sequences classified to the genus level in the NCBI database was vastly greater than both the RDP and SILVA databases combined.

Although each database was predominantly assessed on genus level classifications, 11 ASVs (excluding *C. aminophilum* due to the ambiguous nomenclature of the species) were assigned to the correct species by the RefSeq + RDP database. In addition, *E. ruminantium* was correctly identified at the species level by the RefSeq + RDP database. Complications in the classification of *E. ruminantium* at lower taxonomic levels have been reported with the use of SILVA (Henderson et al., 2019). As explained by the previous authors, this most likely arises due to the sharing of a genus name between taxa belonging to a different family i.e. *E. ruminantium* a member of the *Lachnospiraceae* and *E. limosum* a member of *Eubacteriaceae*.

The GTDB is relatively new in comparison to the RDP, SILVA and RefSeq databases with its curation described in detail by Parks et al. (2018). In short, the database defines bacterial and archaeal taxonomy on the basis of 120 and 122 concatenated protein sequences, respectively (Parks et al., 2017). The previous authors argue the high proportion of metagenome assembled genomes (MAGs) encompassed in many databases, lack 16S rRNA genes due to their repetitive nature. In addition, it is claimed the novel method defined by Parks and colleagues is capable of expanding bacterial and archaeal diversity by 30%.

The GTDB primarily relies on the LPSN for taxonomic classification of sequences (Parks et al., 2018). Therefore, the greater reliance on the LPSN for taxonomic classification by the GTBD may explain some of the variation in the classification of sequences between databases. The LPSN is regularly updated with phylogenetic reclassifications published in the literature. The GTDB (11/11/2018) database available through DADA2 has been more recently updated than the version of SILVA (13/2/2018) and RDP (1/6/2017) used in this study. Therefore, it is possible the GTDB may have contained more recent taxonomies from the LPSN, thus explaining differences in sequence classifications at higher levels between databases. In addition, both the GTDB curators and LPSN have adhered to inclusion of the suffix –ota at the end of the common name of a phylum, as per the recommendation of Whitman et al. (2018).

Previous authors have highlighted the lack of a rumen specific database comparison between the main four 16S rRNA databases (RDP, SILVA, NCBI and GreenGenes) (Li et al., 2018). The GreenGenes reference database clusters sequences on the basis of 97% similarity. As a result, in an effort to adhere to the principles of exact sequence variant analysis as defined by Callahan et al. (2017), reads were not aligned to the GreenGenes database. Similarly, the RIM-DB database was not considered in our analysis as it groups members of the genera *Methanobrevibacter* into clades based on sequence similarity and utilizes unofficial nomenclature for members of the order Methanomassiliicoccales (Seedorf et al., 2014). Furthermore, the RIM-DB has previously been shown to have a reduced capacity to classify *Methanobrevibacter* sequences, to the species level, in comparison to early releases of SILVA (release 111) (Seedorf et al., 2014).

To the best of our knowledge, we believe this to be one of the first comparisons of 16S rRNA database classifications for rumen microbial amplicon analysis that uses a rumen-specific reference standard. Furthermore, this investigation has shown the need for an improvement to the consistency and modernization of taxonomic classifications amongst the main 16S rRNA reference databases. While useful in the comparison of reference databases for microbial community analysis, additional sources of variation which may be present in “true” microbiomes cannot be assessed with the use of a DNA reference standard. For example inhibitors which may impact both DNA extraction and subsequent downstream analysis, such as PCR, are not accounted for with the use of mock communities (Pollock et al., 2018).

As alluded to by others (Park and Won, 2018; Pollock et al., 2018; Henderson et al., 2019), analysis conducted as part of this study highlights the known impact reference database selection can have on results interpretation. However, only a relatively small number of rumen prokaryotic species, in comparison to that which would be represented within the rumen, are included in our reference standard. As a result, discretion is required in the selection of the optimum reference database for rumen metataxonomic studies based on our analysis. With this said, we cautiously identify the RefSeq + RDP and SILVA databases as the most appropriate reference databases for studying the rumen prokaryotic community, when conducting 16S rRNA amplicon sequencing, due to their superior classification of sequences to the genus level. Nonetheless, limitations are apparent with both datasets. For example, both datasets incorrectly classified Methanomassiliicoccales beyond the taxonomic level of order. In addition, while a high number of species classifications occurred with the use of the RefSeq + RDP database, discrepancies in the genus level classification of *Eubacterium* were apparent.

Findings from this study highlight the benefit of utilizing a reference standard, representative of a microbiota that typically inhabits a specific microbial environment under investigation, for the evaluation of database choice for microbial 16S rRNA analysis. In keeping with the recommendations of the MBQC project, we would recommend all microbiome research groups to validate existing, novel and updated versions of all databases for taxonomic classification accuracy, with the use of internal reference standards containing sequences unique and representative of the microbial community under investigation.

## Data Availability Statement

The original contributions presented in the study are publicly available. This data can be found in NCBI under accession number PRJNA674030.

## Author Contributions

MM, SW, and PS conceived and designed the experiments. PS, MM, and CC performed the experiments. PS and MM analyzed the data. RG and HS contributed synthetic microbial DNA and sequence data. PS, MM, and SW interpreted results and drafted the manuscript with input from CC, RG, and HS. All authors contributed to the article and approved the submitted version.

## Conflict of Interest

The authors declare that the research was conducted in the absence of any commercial or financial relationships that could be construed as a potential conflict of interest.

## Footnotes

1 https://benjjneb.github.io/dada2/tutorial.html

2 https://benjjneb.github.io/dada2/training.html

3 https://blast.ncbi.nlm.nih.gov/Blast.cgi?PAGE_TYPE=BlastSearch

4 http://www2.decipher.codes/FindChimeras.html

5 https://lpsn.dsmz.de/genus/lachnoclostridium

## Supplementary Material

Supplementary Table 1 Individual database classification of bacterial (*n* = 13) and archaeal (*n* = 3) sequences included in reference standard (bootstrap = 50).

Supplementary Table 2 Individual database classification of bacterial (*n* = 13) and archaeal (*n* = 3) sequences included in reference standard (bootstrap = 80).

Supplementary Table 3 Average relative abundance of bacterial (*n* = 13) and archaeal (*n* = 3) sequences included in reference standard using the GTDB database (bootstrap = 80).

Supplementary Table 4 Average relative abundance of bacterial (*n* = 13) and archaeal (*n* = 3) sequences included in reference standard using the SILVA database (bootstrap = 80).

Supplementary Table 5 Average relative abundance of bacterial (*n* = 13) and archaeal (*n* = 3) sequences included in reference standard using the RDP database (bootstrap = 80).

Supplementary Table 6 Average relative abundance of bacterial (*n* = 13) and archaeal (*n* = 3) sequences included in reference standard using the Refseq + RDP database (bootstrap = 80).

Supplementary Data 1 Assign taxonomy script for each database.
