# Supplementary material for: Synthetic Sequencing Standards: A Guide to Database Choice for Rumen Microbiota Amplicon Sequencing Analysis
Source: Front Microbiol. 2020 Dec 8;11:606825. doi: 10.3389/fmicb.2020.606825 (PMC7752867; doi:10.3389/fmicb.2020.606825)
Supplement: Supplementary Table 1 — Individual database classification of bacterial (n = 13) and archaeal (n = 3) sequences included in reference standard (bootstrap = 50). [file Data_Sheet_1.pdf]

Supplementary Material

Supp. Table 1 Individual database classification of bacterial (n=13) and archaeal (n=3) sequences included in reference standard (bootstrap=50)

Sequence	Database	Kingdom	Phylum	Class	Order	Family	Genus	Species
1	GTDB	Bacteria	Firmicutes_A	Clostridia	Lachnospirales	Lachnospiraceae	NA	NA
	RDP	Bacteria	Firmicutes	Clostridia	Clostridiales	Lachnospiraceae	NA	NA
	SILVA	Bacteria	Firmicutes	Clostridia	Clostridiales	Lachnospiraceae	NA	NA
	RefSeq + RDP	Bacteria	Firmicutes	Clostridia	Clostridiales	Lachnospiraceae	Lachnospiracea incertae sedis	Eubacterium_ruminantium(AB008552)
2	GTDB	Archaea	Euryarchaeota	Methanobacteria	Methanobacteriales	Methanobacteriaceae	Methanobrevibacter_A	Methanobrevibacter_A_millerae(RS_GCF_001477655.1)
	RDP	Archaea	Euryarchaeota	Methanobacteria	Methanobacteriales	Methanobacteriaceae	Methanobrevibacter	NA
	SILVA	Archaea	Euryarchaeota	Methanobacteria	Methanobacteriales	Methanobacteriaceae	Methanobrevibacter	NA
	RefSeq + RDP	Archaea	Euryarchaeota	Methanobacteria	Methanobacteriales	Methanobacteriaceae	Methanobrevibacter	Methanobrevibacter_millerae(AY196673)
3	GTDB	Archaea	Euryarchaeota	Methanobacteria	Methanobacteriales	Methanobacteriaceae	Methanobrevibacter	Methanobrevibacter_olleyae(RS_GCF_001563245.1)
	RDP	Archaea	Euryarchaeota	Methanobacteria	Methanobacteriales	Methanobacteriaceae	Methanobrevibacter	NA
	SILVA	Archaea	Euryarchaeota	Methanobacteria	Methanobacteriales	Methanobacteriaceae	Methanobrevibacter	NA
	RefSeq + RDP	Archaea	Euryarchaeota	Methanobacteria	Methanobacteriales	Methanobacteriaceae	Methanobrevibacter	Methanobrevibacter_olleyae(AY615201)
4	GTDB	Bacteria	Firmicutes_A	Clostridia	Peptostreptococcales	Peptostreptococcaceae	NA	NA
	RDP	Bacteria	Firmicutes	Clostridia	Clostridiales	Peptostreptococcaceae	Peptostreptococcus	anaerobius
	SILVA	Bacteria	Firmicutes	Clostridia	Clostridiales	Peptostreptococcaceae	Peptostreptococcus	anaerobius
	RefSeq + RDP	Bacteria	Firmicutes	Clostridia	Clostridiales	Peptostreptococcaceae	Peptostreptococcus	Peptostreptococcus_anaerobius(AY326462)
5	GTDB	Bacteria	Firmicutes_A	Clostridia	Lachnospirales	Lachnospiraceae	Pseudobutyrivibrio	Pseudobutyrivibrio_ruminis(RS_GCF_900109715.1)
	RDP	Bacteria	Firmicutes	Clostridia	Clostridiales	Lachnospiraceae	Pseudobutyrivibrio	NA
	SILVA	Bacteria	Firmicutes	Clostridia	Clostridiales	Lachnospiraceae	Pseudobutyrivibrio	NA
	RefSeq + RDP	Bacteria	Firmicutes	Clostridia	Clostridiales	Lachnospiraceae	Pseudobutyrivibrio	Pseudobutyrivibrio_ruminis(X95893)
6	GTDB	Bacteria	Actinobacteriota	Actinobacteria	Propionibacteriales	Propionibacteriaceae	NA	NA
	RDP	Bacteria	Actinobacteria	Actinobacteria	Actinomycetales	Propionibacteriaceae	Propionibacterium	NA
	SILVA	Bacteria	Actinobacteria	Actinobacteria	Propionibacteriales	Propionibacteriaceae	Propionibacterium	australiense
	RefSeq + RDP	Bacteria	Actinobacteria	Actinobacteria	Actinomycetales	Propionibacteriaceae	Propionibacterium	Propionibacterium_australiense(AF225962)
7	GTDB	Bacteria	Firmicutes_A	Clostridia	Oscillospirales	Ruminococcaceae	Ruminococcus_D	Ruminococcus_D_albus(RS_GCF_000179635.2)
	RDP	Bacteria	Firmicutes	Clostridia	Clostridiales	Ruminococcaceae	Ruminococcus	albus
	SILVA	Bacteria	Firmicutes	Clostridia	Clostridiales	Ruminococcaceae	Ruminococcus_1	albus
	RefSeq + RDP	Bacteria	Firmicutes	Clostridia	Clostridiales	Ruminococcaceae	Ruminococcus	Ruminococcus_albus(L76598)
8	GTDB	Bacteria	Firmicutes_A	Clostridia	Lachnospirales	Lachnospiraceae	NA	NA
	RDP	Bacteria	Firmicutes	Clostridia	Clostridiales	Lachnospiraceae	Butyrivibrio	fibrisolvens
	SILVA	Bacteria	Firmicutes	Clostridia	Clostridiales	Lachnospiraceae	Butyrivibrio_2	fibrisolvens
	RefSeq + RDP	Bacteria	Firmicutes	Clostridia	Clostridiales	Lachnospiraceae	Butyrivibrio	Butyrivibrio_fibrisolvens(U41172)
9	GTDB	Bacteria	Firmicutes_C	Negativicutes	Veillonellales	Megasphaeraceae	Megasphaera	Megasphaera_elsdenii(RS_GCF_001304715.1)
	RDP	Bacteria	Firmicutes	Negativicutes	Selenomonadales	Veillonellaceae	Megasphaera	NA
	SILVA	Bacteria	Firmicutes	Negativicutes	Selenomonadales	Veillonellaceae	Megasphaera	NA
	RefSeq + RDP	Bacteria	Firmicutes	Negativicutes	Selenomonadales	Veillonellaceae	Megasphaera	NA
10	GTDB	Archaea	Thermoplasmatota	Thermoplasmata_A	Methanomassiliicoccales	Methanomethylophilaceae	Methanomethylophilus	Methanomethylophilus_alvus(GB_GCA_000437835.1)
	RDP	Archaea	Euryarchaeota	Thermoplasmata	Methanomassiliicoccales	Methanomassiliicoccaceae	Methanomassiliicoccus	NA
	SILVA	Archaea	Euryarchaeota	Thermoplasmata	Methanomassiliicoccales	Methanomethylophilaceae	Candidatus_Methanomethylophilus	NA
	RefSeq + RDP	Archaea	Euryarchaeota	Thermoplasmata	Methanomassiliicoccales	Methanomassiliicoccaceae	Methanomassiliicoccus	Methanomassiliicoccus_luminyensis(HQ896499)
11	GTDB	Bacteria	Firmicutes	Bacilli	Lactobacillales	Streptococcaceae	Streptococcus	Streptococcus_lutetiensis(RS_GCF_001477615.1)

	RDP	Bacteria	Firmicutes	Bacilli	Lactobacillales	Streptococcaceae	Streptococcus	NA
	SILVA	Bacteria	Firmicutes	Bacilli	Lactobacillales	Streptococcaceae	Streptococcus	NA
	RefSeq + RDP	Bacteria	Firmicutes	Bacilli	Lactobacillales	Streptococcaceae	Streptococcus	Streptococcus_infantarius(AF429762)
12	GTDB	Bacteria	Firmicutes_A	Clostridia	Lachnospirales	Lachnospiraceae	Lachnospira	Lachnospira_pectinoschiza_A(RS_GCF_900103815.1)
	RDP	Bacteria	Firmicutes	Clostridia	Clostridiales	Lachnospiraceae	Lachnospira	NA
	SILVA	Bacteria	Firmicutes	Clostridia	Clostridiales	Lachnospiraceae	Lachnospira	NA
	RefSeq + RDP	Bacteria	Firmicutes	Clostridia	Clostridiales	Lachnospiraceae	Lachnospira	Lachnospira_multipara(FR733699)
13	GTDB	Bacteria	Fibrobacterota	Fibrobacteria	Fibrobacterales	Fibrobacteraceae	Fibrobacter	Fibrobacter_succinogenes(RS_GCF_000024665.1)
	RDP	Bacteria	Fibrobacteres	Fibrobacteria	Fibrobacterales	Fibrobacteraceae	Fibrobacter	succinogenes
	SILVA	Bacteria	Fibrobacteres	Fibrobacteria	Fibrobacterales	Fibrobacteraceae	Fibrobacter	succinogenes
	RefSeq + RDP	Bacteria	Fibrobacteres	Fibrobacteria	Fibrobacterales	Fibrobacteraceae	Fibrobacter	Fibrobacter_succinogenes(AJ496032)
14	GTDB	Bacteria	Firmicutes_A	Clostridia	Lachnospirales	Lachnospiraceae	NA	NA
	RDP	Bacteria	Firmicutes	Clostridia	Clostridiales	Lachnospiraceae	Butyrivibrio	NA
	SILVA	Bacteria	Firmicutes	Clostridia	Clostridiales	Lachnospiraceae	Lachnoclostridium_1	NA
	RefSeq + RDP	Bacteria	Firmicutes	Clostridia	Clostridiales	Lachnospiraceae	Clostridium XlVa	Clostridium_aminophilum(L04165)
15	GTDB	Bacteria	Firmicutes_C	Negativicutes	Selenomonadales	Selenomonadaceae	Selenomonas_A	Selenomonas_A_ruminantium_E(RS_GCF_900100835.1)
	RDP	Bacteria	Firmicutes	Negativicutes	Selenomonadales	Veillonellaceae	Selenomonas	ruminantium
	SILVA	Bacteria	Firmicutes	Negativicutes	Selenomonadales	Veillonellaceae	Selenomonas_1	ruminantium
	RefSeq + RDP	Bacteria	Firmicutes	Negativicutes	Selenomonadales	Veillonellaceae	Selenomonas	Selenomonas_ruminantium(M62702)
16	GTDB	Bacteria	Bacteroidota	Bacteroidia	Bacteroidales	Bacteroidaceae	Prevotella	NA
	RDP	Bacteria	Bacteroidetes	Bacteroidia	Bacteroidales	Prevotellaceae	Prevotella	ruminicola
	SILVA	Bacteria	Bacteroidetes	Bacteroidia	Bacteroidales	Prevotellaceae	Prevotella_1	ruminicola
	RefSeq + RDP	Bacteria	Bacteroidetes	Bacteroidia	Bacteroidales	Prevotellaceae	Prevotella	Prevotella_ruminicola(L16482)

Supp. Table 2 Individual database classification of bacterial (n=13) and archaeal (n=3) sequences included in reference standard (bootstrap=80)

Sequence	Database	Kingdom	Phylum	Class	Order	Family	Genus	Species
1	GTDB	Bacteria	Firmicutes_A	Clostridia	Lachnospirales	Lachnospiraceae	NA	NA
	RDP	Bacteria	Firmicutes	Clostridia	Clostridiales	Lachnospiraceae	NA	NA
	SILVA	Bacteria	Firmicutes	Clostridia	Clostridiales	Lachnospiraceae	NA	NA
	RefSeq + RDP	Bacteria	Firmicutes	Clostridia	Clostridiales	Lachnospiraceae	Lachnospiracea incertae sedis	Eubacterium_ruminantium(AB008552)
2	GTDB	Archaea	Euryarchaeota	Methanobacteria	Methanobacteriales	Methanobacteriaceae	Methanobrevibacter_A	Methanobrevibacter_A_millerae(RS_GCF_001477655.1)
	RDP	Archaea	Euryarchaeota	Methanobacteria	Methanobacteriales	Methanobacteriaceae	Methanobrevibacter	NA
	SILVA	Archaea	Euryarchaeota	Methanobacteria	Methanobacteriales	Methanobacteriaceae	Methanobrevibacter	NA
	RefSeq + RDP	Archaea	Euryarchaeota	Methanobacteria	Methanobacteriales	Methanobacteriaceae	Methanobrevibacter	Methanobrevibacter_millerae(AY196673)
3	GTDB	Archaea	Euryarchaeota	Methanobacteria	Methanobacteriales	Methanobacteriaceae	Methanobrevibacter	Methanobrevibacter_olleyae(RS_GCF_001563245.1)
	RDP	Archaea	Euryarchaeota	Methanobacteria	Methanobacteriales	Methanobacteriaceae	Methanobrevibacter	NA
	SILVA	Archaea	Euryarchaeota	Methanobacteria	Methanobacteriales	Methanobacteriaceae	Methanobrevibacter	NA
	RefSeq + RDP	Archaea	Euryarchaeota	Methanobacteria	Methanobacteriales	Methanobacteriaceae	Methanobrevibacter	Methanobrevibacter_olleyae(AY615201)
4	GTDB	Bacteria	Firmicutes_A	Clostridia	Peptostreptococcales	Peptostreptococcaceae	NA	NA
	RDP	Bacteria	Firmicutes	Clostridia	Clostridiales	Peptostreptococcaceae	Peptostreptococcus	anaerobius
	SILVA	Bacteria	Firmicutes	Clostridia	Clostridiales	Peptostreptococcaceae	Peptostreptococcus	anaerobius
	RefSeq + RDP	Bacteria	Firmicutes	Clostridia	Clostridiales	Peptostreptococcaceae	Peptostreptococcus	Peptostreptococcus_anaerobius(AY326462)
5	GTDB	Bacteria	Firmicutes_A	Clostridia	Lachnospirales	Lachnospiraceae	Pseudobutyrivibrio	Pseudobutyrivibrio_ruminis(RS_GCF_900109715.1)
	RDP	Bacteria	Firmicutes	Clostridia	Clostridiales	Lachnospiraceae	Pseudobutyrivibrio	NA
	SILVA	Bacteria	Firmicutes	Clostridia	Clostridiales	Lachnospiraceae	Pseudobutyrivibrio	NA
	RefSeq + RDP	Bacteria	Firmicutes	Clostridia	Clostridiales	Lachnospiraceae	Pseudobutyrivibrio	Pseudobutyrivibrio_ruminis(X95893)
6	GTDB	Bacteria	Actinobacteriota	Actinobacteria	Propionibacteriales	Propionibacteriaceae	Propionibacterium	NA
	RDP	Bacteria	Actinobacteria	Actinobacteria	Actinomycetales	Propionibacteriaceae	Propionibacterium	NA
	SILVA	Bacteria	Actinobacteria	Actinobacteria	Propionibacteriales	Propionibacteriaceae	Propionibacterium	australiense
	RefSeq + RDP	Bacteria	Actinobacteria	Actinobacteria	Actinomycetales	Propionibacteriaceae	Propionibacterium	NA
7	GTDB	Bacteria	Firmicutes_A	Clostridia	Oscillospirales	Ruminococcaceae	Ruminococcus_D	Ruminococcus_D_albus(RS_GCF_000179635.2)
	RDP	Bacteria	Firmicutes	Clostridia	Clostridiales	Ruminococcaceae	Ruminococcus	albus
	SILVA	Bacteria	Firmicutes	Clostridia	Clostridiales	Ruminococcaceae	Ruminococcus_1	albus
	RefSeq + RDP	Bacteria	Firmicutes	Clostridia	Clostridiales	Ruminococcaceae	Ruminococcus	Ruminococcus_albus(L76598)
8	GTDB	Bacteria	Firmicutes_A	Clostridia	Lachnospirales	Lachnospiraceae	NA	NA
	RDP	Bacteria	Firmicutes	Clostridia	Clostridiales	Lachnospiraceae	Butyrivibrio	fibrisolvens
	SILVA	Bacteria	Firmicutes	Clostridia	Clostridiales	Lachnospiraceae	Butyrivibrio_2	fibrisolvens
	RefSeq + RDP	Bacteria	Firmicutes	Clostridia	Clostridiales	Lachnospiraceae	Butyrivibrio	Butyrivibrio_fibrisolvens(U41172)
9	GTDB	Bacteria	Firmicutes_C	Negativicutes	Veillonellales	Megasphaeraceae	Megasphaera	Megasphaera_elsdenii(RS_GCF_001304715.1)
	RDP	Bacteria	Firmicutes	Negativicutes	Selenomonadales	Veillonellaceae	Megasphaera	NA
	SILVA	Bacteria	Firmicutes	Negativicutes	Selenomonadales	Veillonellaceae	Megasphaera	NA
	RefSeq + RDP	Bacteria	Firmicutes	Negativicutes	Selenomonadales	Veillonellaceae	Megasphaera	NA
10	GTDB	Archaea	Thermoplasmatota	Thermoplasmata_A	Methanomassiliicoccales	Methanomethylophilaceae	Methanomethylophilus	Methanomethylophilus_alvus(GB_GCA_000437835.1)
	RDP	Archaea	Euryarchaeota	Thermoplasmata	Methanomassiliicoccales	Methanomassiliicoccaceae	Methanomassiliicoccus	NA
	SILVA	Archaea	Euryarchaeota	Thermoplasmata	Methanomassiliicoccales	Methanomethylophilaceae	Candidatus_Methanomethylophilus	NA
	RefSeq + RDP	Archaea	Euryarchaeota	Thermoplasmata	NA	NA	NA	NA
11	GTDB	Bacteria	Firmicutes	Bacilli	Lactobacillales	Streptococcaceae	Streptococcus	Streptococcus_lutetiensis(RS_GCF_001477615.1)
	RDP	Bacteria	Firmicutes	Bacilli	Lactobacillales	Streptococcaceae	Streptococcus	NA
	SILVA	Bacteria	Firmicutes	Bacilli	Lactobacillales	Streptococcaceae	Streptococcus	NA
	RefSeq + RDP	Bacteria	Firmicutes	Bacilli	Lactobacillales	Streptococcaceae	Streptococcus	Streptococcus_infantarius(AF429762)
12	GTDB	Bacteria	Firmicutes_A	Clostridia	Lachnospirales	Lachnospiraceae	Lachnospira	Lachnospira_pectinoschiza_A(RS_GCF_900103815.1)
	RDP	Bacteria	Firmicutes	Clostridia	Clostridiales	Lachnospiraceae	Lachnospira	NA

	SILVA	Bacteria	Firmicutes	Clostridia	Clostridiales	Lachnospiraceae	Lachnospira	NA
	RefSeq + RDP	Bacteria	Firmicutes	Clostridia	Clostridiales	Lachnospiraceae	Lachnospira	Lachnospira_multipara(FR733699)
13	GTDB	Bacteria	Fibrobacterota	Fibrobacteria	Fibrobacterales	Fibrobacteraceae	Fibrobacter	Fibrobacter_succinogenes(RS_GCF_000024665.1)
	RDP	Bacteria	Fibrobacteres	Fibrobacteria	Fibrobacterales	Fibrobacteraceae	Fibrobacter	succinogenes
	SILVA	Bacteria	Fibrobacteres	Fibrobacteria	Fibrobacterales	Fibrobacteraceae	Fibrobacter	succinogenes
	RefSeq + RDP	Bacteria	Fibrobacteres	Fibrobacteria	Fibrobacterales	Fibrobacteraceae	Fibrobacter	Fibrobacter_succinogenes(AJ496032)
14	GTDB	Bacteria	Firmicutes_A	Clostridia	Lachnospirales	Lachnospiraceae	NA	NA
	RDP	Bacteria	Firmicutes	Clostridia	Clostridiales	Lachnospiraceae	NA	NA
	SILVA	Bacteria	Firmicutes	Clostridia	Clostridiales	Lachnospiraceae	Lachnoclostridium_1	NA
	RefSeq + RDP	Bacteria	Firmicutes	Clostridia	Clostridiales	Lachnospiraceae	Clostridium XlVa	Clostridium_aminophilum(L04165)
15	GTDB	Bacteria	Firmicutes_C	Negativicutes	Selenomonadales	Selenomonadaceae	Selenomonas_A	Selenomonas_A_ruminantium_E(RS_GCF_900100835.1)
	RDP	Bacteria	Firmicutes	Negativicutes	Selenomonadales	Veillonellaceae	Selenomonas	ruminantium
	SILVA	Bacteria	Firmicutes	Negativicutes	Selenomonadales	Veillonellaceae	Selenomonas_1	ruminantium
	RefSeq + RDP	Bacteria	Firmicutes	Negativicutes	Selenomonadales	Veillonellaceae	Selenomonas	Selenomonas_ruminantium(M62702)
16	GTDB	Bacteria	Bacteroidota	Bacteroidia	Bacteroidales	Bacteroidaceae	Prevotella	NA
	RDP	Bacteria	Bacteroidetes	Bacteroidia	Bacteroidales	Prevotellaceae	Prevotella	ruminicola
	SILVA	Bacteria	Bacteroidetes	Bacteroidia	Bacteroidales	Prevotellaceae	Prevotella_1	ruminicola
	RefSeq + RDP	Bacteria	Bacteroidetes	Bacteroidia	Bacteroidales	Prevotellaceae	Prevotella	Prevotella_ruminicola(L16482)

Supp. Table 3 Average relative abundance of bacterial (n=13) and archaeal (n=3) sequences included in reference standard using the GTDB database (bootstrap=80)

Sequence	Kingdom	Phylum	Class	Order	Family	Genus	Species	Run 1	Run 2	Run 3	Mean
1	Bacteria	Firmicutes_A	Clostridia	Lachnospirales	Lachnospiraceae	NA	NA	12.53%	13.02%	10.31%	11.95%
2	Archaea	Euryarchaeota	Methanobacteria	Methanobacteriales	Methanobacteriaceae	Methanobrevibacter_A	Methanobrevibacter_A_millerae(RS_GCF_001477655.1)	10.91%	9.97%	13.83%	11.57%
3	Archaea	Euryarchaeota	Methanobacteria	Methanobacteriales	Methanobacteriaceae	Methanobrevibacter	Methanobrevibacter_olleyae(RS_GCF_001563245.1)	8.05%	7.55%	9.63%	8.41%
4	Bacteria	Firmicutes_A	Clostridia	Peptostreptococcales	Peptostreptococcaceae	NA	NA	7.85%	7.66%	6.88%	7.46%
11	Bacteria	Firmicutes_A	Clostridia	Lachnospirales	Lachnospiraceae	Pseudobutyrivibrio	Pseudobutyrivibrio_ruminis(RS_GCF_900109715.1)	5.35%	7.53%	8.07%	6.98%
6	Bacteria	Actinobacteriota	Actinobacteria	Propionibacteriales	Propionibacteriaceae	Propionibacterium	NA	6.69%	7.09%	6.81%	6.86%
5	Bacteria	Firmicutes_A	Clostridia	Oscillospirales	Ruminococcaceae	Ruminococcus_D	Ruminococcus_D_albus(RS_GCF_000179635.2)	6.95%	7.37%	5.85%	6.72%
7	Bacteria	Firmicutes_A	Clostridia	Lachnospirales	Lachnospiraceae	NA	NA	6.31%	6.80%	5.58%	6.23%
9	Bacteria	Firmicutes_C	Negativicutes	Veillonellales	Megasphaeraceae	Megasphaera	Megasphaera_elsdenii(RS_GCF_001304715.1)	6.06%	6.06%	6.29%	6.14%
8	Archaea	Thermoplasmatota	Thermoplasmata_A	Methanomassiliicoccales	Methanomethylophilaceae	Methanomethylophilus	Methanomethylophilus_alvus(GB_GCA_000437835.1)	6.18%	5.00%	6.73%	5.97%
10	Bacteria	Firmicutes	Bacilli	Lactobacillales	Streptococcaceae	Streptococcus	Streptococcus_lutetiensis(RS_GCF_001477615.1)	5.72%	5.73%	5.36%	5.60%
12	Bacteria	Firmicutes_A	Clostridia	Lachnospirales	Lachnospiraceae	Lachnospira	Lachnospira_pectinoschiza_A(RS_GCF_900103815.1)	5.04%	5.09%	4.13%	4.76%
13	Bacteria	Fibrobacterota	Fibrobacteria	Fibrobacterales	Fibrobacteraceae	Fibrobacter	Fibrobacter_succinogenes(RS_GCF_000024665.1)	3.57%	3.28%	3.23%	3.36%
14	Bacteria	Firmicutes_A	Clostridia	Lachnospirales	Lachnospiraceae	NA	NA	2.96%	2.93%	2.54%	2.81%
15	Bacteria	Firmicutes_C	Negativicutes	Selenomonadales	Selenomonadaceae	Selenomonas_A	Selenomonas_A_ruminantium_E(RS_GCF_900100835.1)	2.93%	3.02%	2.17%	2.71%
16	Bacteria	Bacteroidota	Bacteroidia	Bacteroidales	Bacteroidaceae	Prevotella	NA	2.03%	1.65%	2.18%	1.95%
							Other	0.88%	0.25%	0.42%	0.52%

Supp. Table 4 Average relative abundance of bacterial (n=13) and archaeal (n=3) sequences included in reference standard using the SILVA database (bootstrap=80)

Sequence	Kingdom	Phylum	Class	Order	Family	Genus	Species	Run 1	Run 2	Run 3	Mean
1	Bacteria	Firmicutes	Clostridia	Clostridiales	Lachnospiraceae	NA	NA	12.53%	13.03%	10.31%	11.96%
2	Archaea	Euryarchaeota	Methanobacteria	Methanobacteriales	Methanobacteriaceae	Methanobrevibacter	NA	10.91%	9.97%	13.83%	11.57%
3	Archaea	Euryarchaeota	Methanobacteria	Methanobacteriales	Methanobacteriaceae	Methanobrevibacter	NA	8.05%	7.55%	9.64%	8.41%
4	Bacteria	Firmicutes	Clostridia	Clostridiales	Peptostreptococcaceae	Peptostreptococcus	anaerobius	7.85%	7.66%	6.88%	7.47%
5	Bacteria	Firmicutes	Clostridia	Clostridiales	Ruminococcaceae	Ruminococcus_1	albus	6.95%	7.37%	5.85%	6.72%
6	Bacteria	Actinobacteria	Actinobacteria	Propionibacteriales	Propionibacteriaceae	Propionibacterium	australiense	6.69%	7.09%	6.81%	6.86%
7	Bacteria	Firmicutes	Clostridia	Clostridiales	Lachnospiraceae	Butyrivibrio_2	fibrisolvens	6.31%	6.80%	5.58%	6.23%
8	Archaea	Euryarchaeota	Thermoplasmata	Methanomassiliicoccales	Methanomethylophilaceae	Candidatus_Methanomethylophilus	NA	6.18%	5.00%	6.73%	5.97%
9	Bacteria	Firmicutes	Negativicutes	Selenomonadales	Veillonellaceae	Megasphaera	NA	6.06%	6.06%	6.30%	6.14%
10	Bacteria	Firmicutes	Bacilli	Lactobacillales	Streptococcaceae	Streptococcus	NA	5.72%	5.73%	5.36%	5.60%
11	Bacteria	Firmicutes	Clostridia	Clostridiales	Lachnospiraceae	Pseudobutyrivibrio	NA	5.35%	7.53%	8.08%	6.99%
12	Bacteria	Firmicutes	Clostridia	Clostridiales	Lachnospiraceae	Lachnospira	NA	5.04%	5.09%	4.13%	4.76%
13	Bacteria	Fibrobacteres	Fibrobacteria	Fibrobacterales	Fibrobacteraceae	Fibrobacter	succinogenes	3.57%	3.28%	3.23%	3.36%
14	Bacteria	Firmicutes	Clostridia	Clostridiales	Lachnospiraceae	Lachnoclostridium_1	NA	2.96%	2.93%	2.54%	2.81%
15	Bacteria	Firmicutes	Negativicutes	Selenomonadales	Veillonellaceae	Selenomonas_1	ruminantium	2.93%	3.02%	2.17%	2.71%
16	Bacteria	Bacteroidetes	Bacteroidia	Bacteroidales	Prevotellaceae	Prevotella_1	ruminicola	2.03%	1.66%	2.18%	1.95%
							Other	0.87%	0.23%	0.38%	0.49%

Supp. Table 5 Average relative abundance of bacterial (n=13) and archaeal (n=3) sequences included in reference standard using the RDP database (bootstrap=80)

Sequence	Kingdom	Phylum	Class	Order	Family	Genus	Species	Run 1	Run 2	Run 3	Mean
1	Bacteria	Firmicutes	Clostridia	Clostridiales	Lachnospiraceae	NA	NA	12.53%	13.02%	10.31%	11.96%
2	Archaea	Euryarchaeota	Methanobacteria	Methanobacteriales	Methanobacteriaceae	Methanobrevibacter	NA	10.91%	9.97%	13.83%	11.57%
3	Archaea	Euryarchaeota	Methanobacteria	Methanobacteriales	Methanobacteriaceae	Methanobrevibacter	NA	8.05%	7.55%	9.63%	8.41%
4	Bacteria	Firmicutes	Clostridia	Clostridiales	Peptostreptococcaceae	Peptostreptococcus	anaerobius	7.85%	7.66%	6.88%	7.47%
11	Bacteria	Firmicutes	Clostridia	Clostridiales	Lachnospiraceae	Pseudobutyrivibrio	NA	5.35%	7.53%	8.07%	6.98%
6	Bacteria	Actinobacteria	Actinobacteria	Actinomycetales	Propionibacteriaceae	Propionibacterium	NA	6.69%	7.09%	6.81%	6.86%
5	Bacteria	Firmicutes	Clostridia	Clostridiales	Ruminococcaceae	Ruminococcus	albus	6.95%	7.37%	5.85%	6.72%
7	Bacteria	Firmicutes	Clostridia	Clostridiales	Lachnospiraceae	Butyrivibrio	fibrisolvens	6.31%	6.80%	5.58%	6.23%
9	Bacteria	Firmicutes	Negativicutes	Selenomonadales	Veillonellaceae	Megasphaera	NA	6.06%	6.06%	6.29%	6.14%
8	Archaea	Euryarchaeota	Thermoplasmata	Methanomassiliicoccales	Methanomassiliicoccaceae	Methanomassiliicoccus	NA	6.18%	5.00%	6.73%	5.97%
10	Bacteria	Firmicutes	Bacilli	Lactobacillales	Streptococcaceae	Streptococcus	NA	5.72%	5.73%	5.36%	5.60%
12	Bacteria	Firmicutes	Clostridia	Clostridiales	Lachnospiraceae	Lachnospira	NA	5.04%	5.09%	4.13%	4.76%
13	Bacteria	Fibrobacteres	Fibrobacteria	Fibrobacterales	Fibrobacteraceae	Fibrobacter	succinogenes	3.57%	3.28%	3.23%	3.36%
14	Bacteria	Firmicutes	Clostridia	Clostridiales	Lachnospiraceae	NA	NA	2.96%	2.93%	2.54%	2.81%
15	Bacteria	Firmicutes	Negativicutes	Selenomonadales	Veillonellaceae	Selenomonas	ruminantium	2.93%	3.02%	2.17%	2.71%
16	Bacteria	Bacteroidetes	Bacteroidia	Bacteroidales	Prevotellaceae	Prevotella	ruminicola	2.03%	1.65%	2.18%	1.95%
							Other	0.87%	0.25%	0.41%	0.50%

Supp. Table 6 Average relative abundance of bacterial (n=13) and archaeal (n=3) sequences included in reference standard using the Refseq + RDP database (bootstrap=80)

Sequence	Kingdom	Phylum	Class	Order	Family	Genus	Species	Run 1	Run 2	Run 3	Mean
1	Bacteria	Firmicutes	Clostridia	Clostridiales	Lachnospiraceae	Lachnospiracea incertae sedis	Eubacterium_ruminantium(AB008552)	12.53%	13.02%	10.31%	11.96%
2	Archaea	Euryarchaeota	Methanobacteria	Methanobacteriales	Methanobacteriaceae	Methanobrevibacter	Methanobrevibacter_millerae(AY196673)	10.91%	9.97%	13.83%	11.57%
3	Archaea	Euryarchaeota	Methanobacteria	Methanobacteriales	Methanobacteriaceae	Methanobrevibacter	Methanobrevibacter_olleyae(AY615201)	8.05%	7.55%	9.63%	8.41%
4	Bacteria	Firmicutes	Clostridia	Clostridiales	Peptostreptococcaceae	Peptostreptococcus	Peptostreptococcus_anaerobius(AY326462)	7.85%	7.66%	6.88%	7.46%
5	Bacteria	Firmicutes	Clostridia	Clostridiales	Ruminococcaceae	Ruminococcus	Ruminococcus_albus(L76598)	6.95%	7.37%	5.85%	6.72%
6	Bacteria	Actinobacteria	Actinobacteria	Actinomycetales	Propionibacteriaceae	Propionibacterium	NA	6.69%	7.09%	6.81%	6.86%
7	Bacteria	Firmicutes	Clostridia	Clostridiales	Lachnospiraceae	Butyrivibrio	Butyrivibrio_fibrisolvens(U41172)	6.31%	6.80%	5.58%	6.23%
8	Archaea	Euryarchaeota	Thermoplasmata	NA	NA	NA	NA	6.18%	5.00%	6.73%	5.97%
9	Bacteria	Firmicutes	Negativicutes	Selenomonadales	Veillonellaceae	Megasphaera	NA	6.06%	6.06%	6.29%	6.14%
10	Bacteria	Firmicutes	Bacilli	Lactobacillales	Streptococcaceae	Streptococcus	Streptococcus_infantarius(AF429762)	5.72%	5.73%	5.36%	5.60%
11	Bacteria	Firmicutes	Clostridia	Clostridiales	Lachnospiraceae	Pseudobutyrivibrio	Pseudobutyrivibrio_ruminis(X95893)	5.35%	7.53%	8.07%	6.98%
12	Bacteria	Firmicutes	Clostridia	Clostridiales	Lachnospiraceae	Lachnospira	Lachnospira_multipara(FR733699)	5.04%	5.09%	4.13%	4.76%
13	Bacteria	Fibrobacteres	Fibrobacteria	Fibrobacterales	Fibrobacteraceae	Fibrobacter	Fibrobacter_succinogenes(AJ496032)	3.57%	3.28%	3.23%	3.36%
14	Bacteria	Firmicutes	Clostridia	Clostridiales	Lachnospiraceae	Clostridium XlVa	Clostridium_aminophilum(L04165)	2.96%	2.93%	2.54%	2.81%
15	Bacteria	Firmicutes	Negativicutes	Selenomonadales	Veillonellaceae	Selenomonas	Selenomonas_ruminantium(M62702)	2.93%	3.02%	2.17%	2.71%
16	Bacteria	Bacteroidetes	Bacteroidia	Bacteroidales	Prevotellaceae	Prevotella	Prevotella_ruminicola(L16482)	2.03%	1.65%	2.18%	1.95%
							Other	0.87%	0.25%	0.41%	0.51%

Supp. Data 1 Assign taxonomy script for each database

Bootstrap threshold (50)

GTDB

```
taxa <- assignTaxonomy(X, "~/GTDB_bac-arc_ssu_r86.fa.gz", multithread=TRUE)
```

RDP

```
taxa <- assignTaxonomy(X, "~/ rdp_train_set_16.fa.gz", multithread=TRUE)
taxa <- addSpecies(taxa80, "~/ rdp_species_assignment_16.fa.gz")
```

SILVA

```
taxa <- assignTaxonomy(X, "~/silva_nr_v132_train_set.fa.gz", multithread=TRUE)
taxa <- addSpecies(taxa80, "~/silva_species_assignment_v132.fa.gz")
```

RefSeq + RDP

```
taxa <- assignTaxonomy(X, " RefSeq-RDP16S_v2_May2018.fa.gz", multithread=TRUE)
```

Bootstrap threshold (80)

GTDB

```
taxa80 <- assignTaxonomy(X, "~/GTDB_bac-arc_ssu_r86.fa.gz", multithread=TRUE, minBoot=80)
```

RDP

```
taxa80 <- assignTaxonomy(X, "~/ rdp_train_set_16.fa.gz", multithread=TRUE, minBoot=80)
taxa <- addSpecies(taxa80, "~/ rdp_species_assignment_16.fa.gz")
```

SILVA

```
taxa80 <- assignTaxonomy(X, "~/silva_nr_v132_train_set.fa.gz", multithread=TRUE, minBoot=80)
taxa <- addSpecies(taxa80, "~/silva_species_assignment_v132.fa.gz")
```

RefSeq + RDP

```
taxa80 <- assignTaxonomy(X, "~/ RefSeq-RDP16S_v2_May2018.fa.gz", multithread=TRUE, minBoot=80)
```

‘X’ = Chimera free sequence table
